# Tris(2-Aminoethyl)Amine/Metal Oxides Hybrid Materials—Preparation, Characterization and Catalytic Application

**DOI:** 10.3390/molecules25204689

**Published:** 2020-10-14

**Authors:** Katarzyna Stawicka, Maria Ziolek

**Affiliations:** Faculty of Chemistry, Adam Mickiewicz University in Poznan, Uniwersytetu Poznanskiego 8, 61-614 Poznan, Poland; ziolek@amu.edu.pl

**Keywords:** basicity, acidity, mesoporous oxides, TAEA, Knoevenagel condensation

## Abstract

Three different metal oxides (basic MgO, basic-acidic Al_2_O_3_ and acidic-basic Nb_2_O_5_) characterized by comparable surface areas (MgO—130 m^2^/g; Al_2_O_3_—172 m^2^/g and Nb_2_O_5_—123 m^2^/g) and pore systems (domination of mesopores with narrow pore size distribution) were modified with tris(2-aminoethyl)amine (TAEA) via two methods: (i) direct anchoring of amine on metal oxide and (ii) anchoring of amine on metal oxide functionalized with (3-chloropropyl)trimethoxysilane. The obtained hybrid materials were characterized in terms of effectiveness of modifier anchoring (elemental analysis), their structural/textural properties (nitrogen adsorption/desorption, XRD), acidity/basicity of support (2-propanol dehydration and dehydrogenation, dehydration and cyclization of 2,5-hexanedione), states of modifier deposited on supports (XPS, FTIR, UV–VIS) and the strength of interaction between the modifier and the support (TG/DTG). It was evidenced that acidic-basic properties of metal oxides as well as the procedure of modification with TAEA determined the ways of amine anchoring and the strength of its interaction with the support. The obtained hybrid materials were tested in Knoevenagel condensation between furfural and malononitrile. The catalysts based on MgO showed superior activity in this reaction. It was correlated with the way of TAEA anchoring on basic MgO and the strength of modifier anchoring on the support. To the best of our knowledge tris(2-aminoethyl)amine has not been used as a modifier of solid supports for enhancement of the catalyst activity in Knoevenagel condensation.

## 1. Introduction

The aim of this study was to establish the effect of acid-base properties of metal oxides used as supports for tris(2-aminoethyl)amine (TAEA) on the forms of TAEA anchored and activity in Knoevenagel condensation between furfural and malononitrile. Three different commercial metal oxides (MgO, Al_2_O_3_, Nb_2_O_5_) expected to show different acid-base properties were applied for anchoring of TAEA by the use of one step direct modification and two-step TAEA loading after functionalization of metal oxides with (3-chloropropyl)trimethoxysilane (ClPTMS). To the best of our knowledge tris(2-aminoethyl)amine has not been used as a modifier of solid supports for enhancement of the catalyst activity in Knoevenagel condensation.

The Knoevenagel condensation can be successfully performed with the use of different amines as catalysts in the homogeneous [1,2] and heterogeneous [3,4,5,6,7,8,9] systems. The Knoevenagel reaction is the condensation which involves a nucleophilic addition of an active hydrogen compound to a carbonyl group followed by a dehydration reaction in which water is eliminated. Often the product is α,β-unsaturated ketone (a conjugated enone). In this reaction the carbonyl group is a component of an aldehyde or a ketone. It is known that Knoevenagel condensation can be catalysed by acidic or basic centres on the catalyst surface [5], but the later ones are more effective in this reaction. The catalysts are usually weakly basic amines. Using a strong base in this reaction would induce self-condensation of the aldehyde or ketone. The Knoevenagel condensation is one of the most useful and widely employed reactions applied for the synthesis of substituted electrophilic alkenes that are further applied as the organic compounds for the synthesis of polymers, fine chemicals, pharmaceuticals or fluorescent dyes [10,11,12]. Substituted alkenes can act moreover as biological ingredients in anticancer or antimalarial pharmaceutical formulations [13,14].

The most often studied heterogeneous catalytic systems for Knoevenagel condensation include amines (e.g., APTMS—(3-aminopropyl)trimethoxysilane) [5], melamine [15], (3-(2-aminoethylamino)propyl)trimethoxysilane [3]) loaded on different supports such as mesoporous silicas [16], matallosilicates [5], resins [17], metal organic frameworks [18], zeolites [19] or fibres [20]. Amine groups are the active sites in this reaction. The comparison of catalysts functionalized with mono and diamines clearly showed, that diamine-modified samples exhibited better activity in Knoevenagel condensation than catalysts containing modifier with only one amine group. The example could be given by the results published in [21], where the authors synthesized K10 montmorillonite functionalized with (3-aminopropyl)triethoxysilane (APTES) and N-(2-aminoethyl)-3-aminopropyltrimethoxysilane (AAPTMS). The diamine functionalized sample presented a higher activity in Knoevenagel condensation between benzaldehyde and diethyl malonate even at room temperature and under solvent-free conditions for 12 h. The another example is given in [22]. The authors modified MCM-41 with APTES, AAPTMS or (3-pyrrolidin-3-ylurea)propyltriethoxysilane. It is worth noting that the last modifier possess three amine groups. MCM-41 modified with this compound showed the highest yield of ethyl (E)-α-cyanocinnamate in reaction between benzaldehyde and ethyl cyaonacetate among tested catalysts. The described results clearly demonstrated that the catalysts with higher number of free amine groups accelerated the Knoevenagel condensation. Thus, the idea of using tris(2-aminoethyl)amine in this work as the active phase assumes that if a modifier contains three amine groups in one molecule (as is the case of TAEA) instead of one –NH_2_ group in monoamines (e.g., APTMS) or two in diamine (e.g., AAPTMS) the activity of the catalyst should be enhanced. The anchoring of TAEA would depend on the support surface properties and, therefore, metal oxides showing different properties were applied in this work as the modifier supports.

## 2. Results and Discussion

### 2.1. Characterization of Supports

Three different commercial supports i.e., MgO, Al_2_O_3_ and Nb_2_O_5_ were used for tris(2-aminoethyl)amine deposition. The acid-base properties of these selected oxides were characterized in two test reactions, i.e., 2-propanol dehydration and dehydrogenation as well as dehydration and cyclization of 2,5-hexanedione (2,5-HDN). The products formed in these reactions are the indicators of acidity or basicity of the investigated catalysts. It is known that 2-propanol is transformed into propene on acidic active centres (both Lewis and Brønsted types), acetone is obtained on Lewis basic active sites, whereas diisopropyl ether is detected in the presence of both Lewis acidic and Lewis basic active centres [23]. In the case of 2,5-HDN transformation, the substrate is converted into 2,5-dimethylfuran (DMF), if the sample contains Brønsted acid sites (BAS), while 3-methyl-2-cyclopenten-1-one (MCP) is obtained in the presence of Brønsted basic centres [24]. The results of both reactions are discussed below.

**Figure 1:**
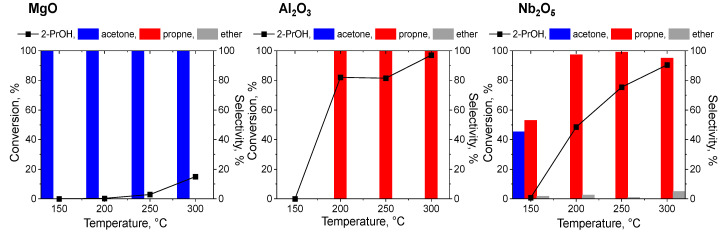
2-Propanol decomposition at 350 °C.

MgO showed the lowest activity in 2-propanol decomposition (Figure 1) and the highest activity in 2,5-hexanedione dehydration and cyclization (Table 1) from among the tested catalysts. In both reactions the obtained products (acetone and MCP, respectively) confirmed the basic character of MgO. The reaction of 2-propanol dehydrogenation to acetone requires the presence of Lewis basic centres, whereas in 2,5-HDN transformation to MCP Brønsted basic centres are involved. This difference in the nature of basic centres active in both test reactions is the reason for very low activity of MgO in 2-propanol reaction and its much higher activity in 2,5-HDN transformation. The highest activity of MgO in 2,5-HDN transformation to MCP pointed to the presence of effective Brønsted basic centres on the MgO surface. When using Al_2_O_3_ and Nb_2_O_5_ as catalysts, 2-PrOH dehydrogenation led mainly to propene, whereas in 2,5-HDN transformation both samples showed a higher selectivity to DMF than MgO. The visibly higher selectivity of Nb_2_O_5_ to DMF than that of Al_2_O_3_ is due to the predominance of Brønsted acidity over basicity on niobium(V) oxide surface. In contrast, in the presence of Al_2_O_3_ the sequence of product distribution was the opposite, MCP dominated in the reaction products pointing to the predominance of Brønsted basic centres on this material. 

In 2,5-HDN dehydration and cyclization, the selectivity ratio of MCP/DMF is an indicator of acidity or basicity of the solid sample surface. If this ratio is above 1 the catalyst is more basic, whereas if it is below 1 the solid sample exhibits more acidic properties. As shown in Table 1, the MCP/DMF ratio for MgO was 249 indicating the basicity of its surface. This ratio was lower for the reaction performed in the presence of Al_2_O_3_. This sample possess more basic than acidic centres. The lowest MCP/DMF ratio was obtained for Nb_2_O_5_, which confirms the acidic properties of this solid, although a low conversion of the substrate suggests rather low concentration of BAS. Moreover, the presence of MCP (34.1% selectivity) in the reaction products indicated that Brønsted basic centres were also present on the niobia surface. Additionally, the presence of pairs of Lewis acidic and basic centres can be concluded from the appearance of diisopropyl ether in the products of 2-propanol bimolecular dehydration.

**Scheme 1:**
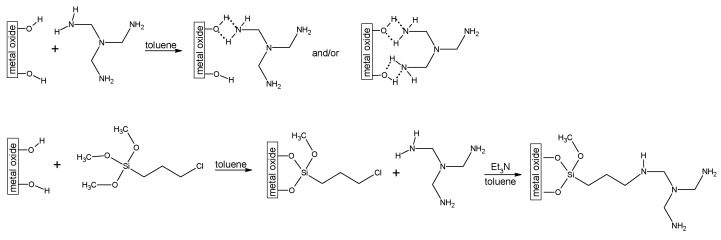
The modification of metal oxides (MgO and Al_2_O_3_) with tris(2-aminoethyl)amine, directly (**upper**) and after ClPTMS (**bottom**).

Acid-base properties of the metal oxide used as a support for TAEA determined the amount of the modifier deposition. Irrespective of the method applied for amine deposition on the metal oxide, both supports containing acidic centres (Al_2_O_3_ and Nb_2_O_5_) anchored more TAEA than basic MgO (Table 2). However, there is a difference between Al_2_O_3_ and Nb_2_O_5_ in direct deposition of TAEA. More TAEA was loaded on Nb_2_O_5_ (sample 3NH_2_ + Nb_2_O_5_) in which acidic sites dominated over basic ones. In contrast, if two-step modification (first functionalization with ClPTMS and next TAEA deposition) was applied, the amount of deposited TAEA was the same for both metal oxides because the first modifier was anchored on both Brønsted basic and acidic centres and TAEA was linked exclusively through anchored ClPTMS (Scheme 1). 

**Figure 2:**
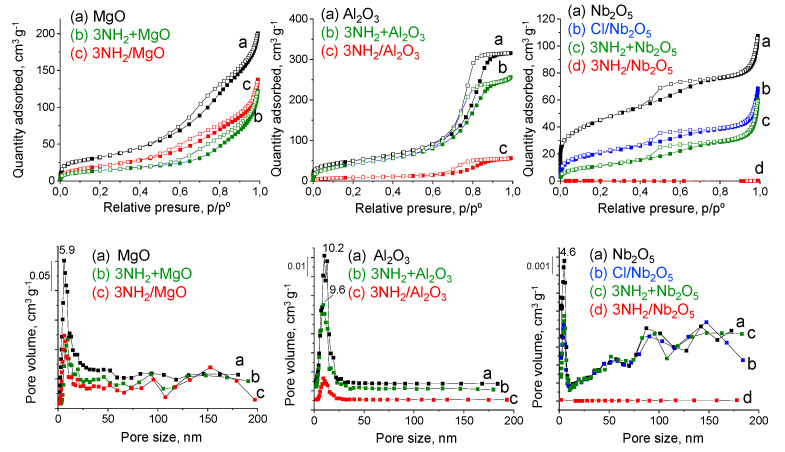
Nitrogen adsorption/desorption isotherms and pore size distribution for metal oxides before and after modification with tris(2-aminoethyl)amine.

The nitrogen adsorption/desorption isotherms of the commercial oxides, shown in Figure 2, are of type IV with hysteresis loop of type H2, according to IUPAC classification. The presence of hysteresis loop is correlated with the existence of mesopores in examined supports, while its type H2 means that the pores have the like ink bottle shape (narrow neck and wide body) [25]. 

Tris(2-aminoethyl)amine anchoring on mesoporous oxides caused changes in the isotherms (independently of the strategy of amine loading). The hysteresis loops were still of type H2 for 3NH_2_/MgO, 3NH_2_/Al_2_O_3_, 3NH_2_ + MgO, 3NH_2_ + Al_2_O_3_ and 3NH_2_ + Nb_2_O_5_, while for 3NH_2_/Nb_2_O_5_ the isotherm was totally collapsed. This suggests that amine blocked the pores in niobium(V) oxide. To verify this hypothesis the nitrogen adsorption/desorption on Cl/Nb_2_O_5_ was performed (Nb_2_O_5_ was modified only with ClPTMS). It was proved that anchoring of ClPTMS before TAEA deposition did not cause significant changes in the shape of the Nb_2_O_5_ isotherm, which confirmed a profound influence of amine on the pores in niobium(V) oxide support. For the other metal oxides, the volume of adsorbed nitrogen also decreased after amine loading, but not as spectacular as for 3NH_2_/Nb_2_O_5_.

The pore size distribution (PSD) presented in Figure 2 is narrow for magnesium oxide and alumina, whereas it is wider for niobium(V) oxide due to the coexistence of mesopores and macropores. The PSD was not changed after TAEA deposition significantly for 3NH_2_/Al_2_O_3_ and 3NH_2_ + Al_2_O_3_, while in 3NH_2_/MgO and 3NH_2_ + MgO macropores appeared. In 3NH_2_/Nb_2_O_5_ no peaks of PSD were observed, most probably due to the blockade of pores in niobium(V) oxide by tris(2-aminoethyl)amine. It was verified by the PSD of Cl/Nb_2_O_5_, which showed a similar pore distribution as Nb_2_O_5_. It is worth noting that PSD of all three metal oxides showed sharp peaks in mesoporous range, indicating the lowest average mesopore size for Nb_2_O_5_ (4.6 nm), medium size for MgO (5.9 nm) and the highest for Al_2_O_3_ (10.2 nm).

The texture parameters of commercial oxides before and after modification with TAEA presented in Table 3, summarize the data obtained from nitrogen adsorption/desorption isotherms. The surface area of commercial supports was in the range of 123–172 m^2^/g, while the diameters of their pores corresponded to mesopores [25]. The anchoring of tris(2-aminoethyl)amine on the surface of mesoporous oxides (independently of the strategy of amine loading) caused a significant reduction of surface area and pore volume of modified samples, especially in the case of 3NH_2_/Nb_2_O_5_. The mentioned parameters decreased after ClPTMS anchoring to Cl/Nb_2_O_5_, however the amine loading on 3NH_2_/Nb_2_O_5_ caused the total blocking of pores in Nb_2_O_5_, and thus the surface area and pore volume of 3NH_2_/Nb_2_O_5_ was reduced almost to zero. The decrease in the surface area and pore volume after TAEA modification confirms the modifier loading inside or at the entrance to the pores of MgO, Al_2_O_3_ and Nb_2_O_5_. 

**Figure 3:**
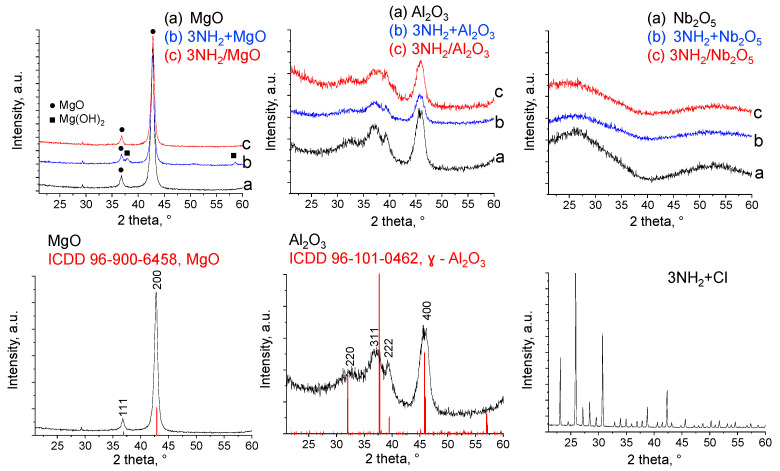
The wide angle XRD patterns of studied materials and organic modifier (3NH_2_ + Cl).

The wide angle XRD patterns (Figure 3) of the metal oxides studied show the diffraction peaks typical of MgO (ICDD 96-900-6458), ɣ-Al_2_O_3_ (ICDD 96-101-0462) and amorphous niobium(V) oxide [26,27,28]. The modification of the supports with TAEA after ClPTMS anchoring did not cause the formation of 3NH_2_ + Cl compound typically generated in the reaction between TAEA and chlorine precursor, if the process was performed in the absence of the support. This conclusion is based on the lack of reflexes characteristic of this compound in Figure 3 for the XRD patterns of 3NH_2_/MgO, 3NH_2_/Al_2_O_3_ and 3NH_2_/Nb_2_O_5_. Both strategies of supports modification with TAEA did not disturb the structure of mesoporous oxides. However, the intensity of reflexes typical of supports decreased after amine loading that confirms the filling of pores with TAEA. Interestingly, in the XRD pattern of 3NH_2_ + MgO the two well resolved reflexes at ca. 38° and 58° of 2 theta were detected, which can be assigned to the Mg(OH)_2_ phase [29]. 

### 2.2. Characterization of TAEA/Metal Oxide Composites 

The tris(2-aminoethyl)amine species introduced into MgO, Al_2_O_3_ and Nb_2_O_5_ supports and the interaction of metal oxide surface species with ClPTMS and TAEA were characterized using FTIR, UV–VIS, XPS and thermogravimetric analyses.

**Figure 4:**
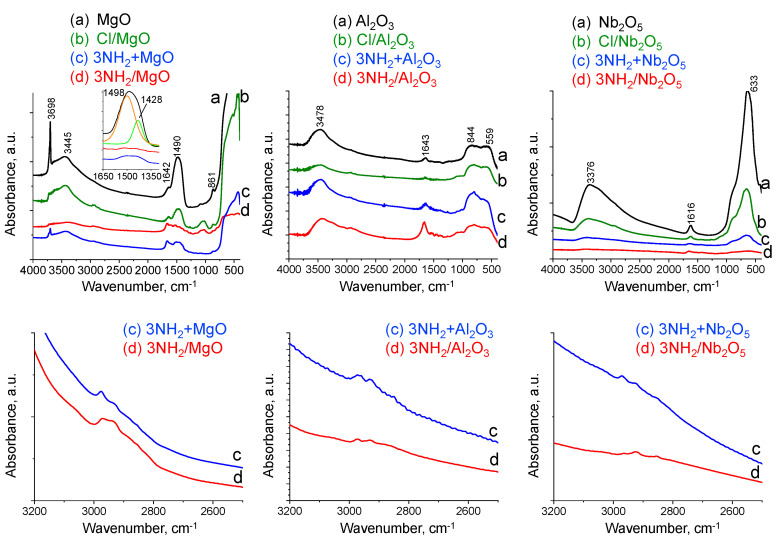
FTIR spectra before and after TAEA loading onto metal oxides. The analysed pills were prepared by mixing of 0.001 g of sample with 0.2 g of KBr.

The FTIR spectra before and after tris(2-aminoethyl)amine anchoring are presented in Figure 4. The spectrum of MgO shows the band typical of Mg-O vibration at 861 cm^−1^ as well as the bands at 3445 cm^−1^ and 1642 cm^−1^ typical of stretching and bending –OH vibrations in adsorbed water molecules. The above-mentioned bands were accompanied by the presence of an intensive band at 3698 cm^−1^ characteristic of Mg-OH vibration [30]. The presence of the bands at 1498 cm^−1^ and 1428 cm^−1^ could be correlated with the interaction of solid MgO with CO_2_ from the air, because these bands are typical of carbonates [16]. 

The modification of MgO with tris(2-aminoethyl)amine caused the appearance of new bands in the range of 3000–2800 cm^−1^. These bands are typical of C-H vibration from TAEA species [31]. Their appearance confirms successful loading of the modifier on MgO support. The intensity of the bands assigned to Mg-OH and Mg-O in the spectrum of pure MgO decreased significantly after TAEA anchoring, independently of the strategy of amine loading. However, the decrease in the bands intensity was higher if amine was anchored into MgO after ClPTMS functionalization. It is clear that basic hydroxyls (Mg-OH) were used in the functionalization of MgO with ClPTMS as evidenced by a decrease in the intensity of the band coming from Mg-OH for Cl/MgO sample. The following decrease in the intensity of the band at ~3700 cm^−1^ observed after TAEA anchoring on ClPTMS-modified magnesium oxide suggested that the amine was anchored not only via ClPTMS but also partially on the rest of Mg-OH. Significantly lower intensity of the bands coming from carbonates indicates that the interaction of MgO covered with TAEA modifier with carbon dioxide from the air towards magnesium carbonate was weaker. Interestingly, in the spectra of Cl/MgO and 3NH_2_/MgO the new band at 1038 cm^−1^ appeared, which corresponds to Si-O-C stretching vibrations from methoxy species from ClPTMS connected to silicon atom (Scheme 1) [31]. This band certifies the presence of ClPTMS in both samples. 

In the FTIR spectrum of Al_2_O_3_, broad bands at ca. 844 cm^−1^ and 559 cm^−1^ were detected, which corresponded to the characteristic vibration of alumina (O-Al-O-Al and Al-OH). The bands detected at 3478 cm^−1^ and 1643 cm^−1^ are typical of the stretching and bending vibration of –OH bonds in adsorbed water [32]. The anchoring of TAEA on the surface of Al_2_O_3_ caused the appearance of new bands in the range of 3000–2800 cm^−1^ as in the spectrum of MgO sample, which confirmed the TAEA presence on the alumina support. Similarly, the intensity of the bands typical of alumina decreased after its modification with TAEA. It is important to stress that Al-OH groups are involved in both, ClPTMS and TAEA anchoring.

In the FTIR spectrum of Nb_2_O_5_, a broad band at 3376 cm^−1^ appeared, which could be assigned to asymmetric and symmetric stretching vibrations of –OH species of water molecules adsorbed on the hydroxyls present on the surface of niobium(V) oxide. The sharp band at 1616 cm^−1^ could be assigned to the bending vibration of water molecules, while the intensive band at 633 cm^−1^ is typical of symmetric stretching vibration in niobium polyhydrate [33].

The intensity of the discussed above bands decreased after TAEA loading for both samples 3NH_2_/Nb_2_O_5_ and 3NH_2_ + Nb_2_O_5_. At the same time, new bands were detected in the range of 3000–2800 cm^−1^ typical of C-H vibration of tris(2-aminoethyl)amine, which verified successful amine loading. The decrease in bands intensity observed for Nb_2_O_5_ after TAEA anchoring was more significant for 3NH_2_/Nb_2_O_5_, which was also modified with ClPTMS before TAEA loading. This phenomenon is due to much larger covering of niobia surface with modifiers if two-step modification was applied. 

**Figure 5:**
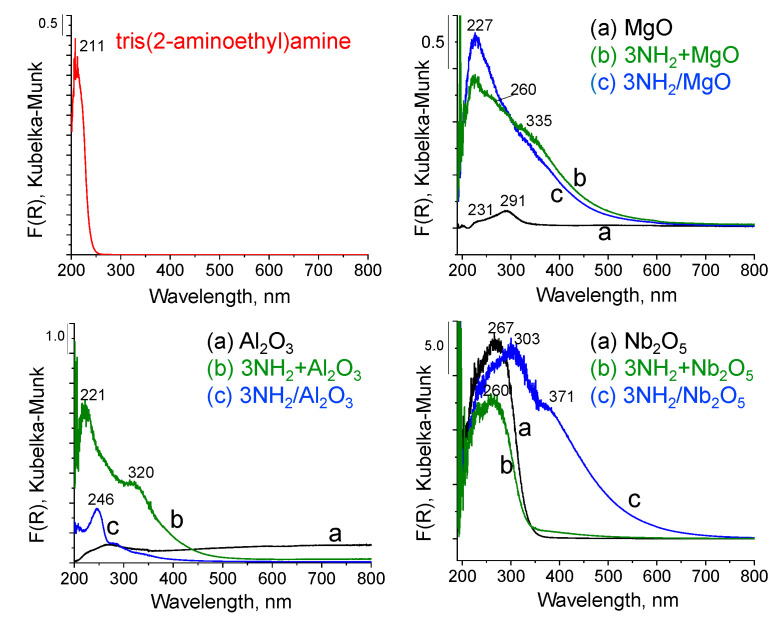
UV–VIS spectra of tris(2-aminoethyl)amine, metal oxide supports and modified samples.

UV–VIS spectroscopy investigations (Figure 5) have brought information about differences in TAEA anchoring depending on the modification procedure. The UV–VIS spectrum of TAEA shows a band at 211 nm. The anchoring of amine directly on the surface of metal oxides caused the appearance of new bands in the UV–VIS spectra. Two bands typical of TAEA anchored on metal oxides were well resolved in the spectrum of 3NH_2_ + Al_2_O_3_ and less resolved but clearly marked in the spectrum of 3NH_2_ + MgO (Figure 5, spectra (b)). The band typical of original amine was red-shifted to longer wavelength, which proved a strong interaction between the modifier and metal oxides. Such a bathochromic shift occurs if the functional group (amine) losses proton releasing an extra electron pair, which takes part in the anchoring of TAEA on the metal oxide surface. Basic hydroxyls present on MgO and Al_2_O_3_ (as documented by the test reaction—Table 1) are involved in proton abstraction from amine group and the electron transition of the ligand-to-metal charge transfer (LMCT), which results in the appearance of a UV band at 227 nm for 3NH_2_ + MgO and at 221 nm for 3NH_2_ + Al_2_O_3_. In addition to the mentioned bands, another band above 300 nm appeared after amine deposition. This suggests that amine was heterogeneously anchored on the surface of both supports. The bands at 335 nm for 3NH_2_ + MgO and at 320 nm for 3NH_2_ + Al_2_O_3_ are supposed to originate from TAEA double coordinated to metal oxide surface via two hydroxyl groups from the solid surface and two amine groups from TAEA as shown in Scheme 1.

The character of the spectrum of 3NH_2_ + Nb_2_O_5_ sample is different. The band characteristic of TAEA anchored on niobium(V) oxide could be overlapped by the bands typical of niobium in tetrahedral (ca. 210 nm) and pentahedral (ca. 250 nm) coordination [34] and, therefore, one cannot conclude about the way TAEA anchors on the basis of this spectrum. If TAEA was anchored on niobium(V) oxide functionalized by ClPTMS two new bands at 303 nm and 371 nm are well-resolved (sample 3NH_2_/Nb_2_O_5_). We propose that they characterize changes in niobium coordination that resulted from ClPTMS anchoring (the first one) and anchoring of TAEA directly on niobium Lewis acid sites via coordinative bond between nitrogen in amine groups and LAS (the band at 371 nm resulted from the LMCT). 

In contrast to niobium(V) oxide modified by functionalization with ClPTMS followed by TAEA deposition, the same modification procedure used for alumina and magnesium oxide resulted in the appearance of one maximum in their UV–VIS spectra (at 227 nm for 3NH_2_/MgO and at 246 nm for 3NH_2_/Al_2_O_3_). However, for 3NH_2_/MgO one cannot exclude the presence of the second band at ca. 330 nm which could be covered by a long tail in the spectrum (Figure 5 spectrum (c)). Thus, homogeneous anchoring of TAEA via functionalization with ClPTMS can be concluded mainly for 3NH_2_/Al_2_O_3_ and supposed for 3NH_2_/MgO, as illustrated in Scheme 1. 

**Figure 6:**
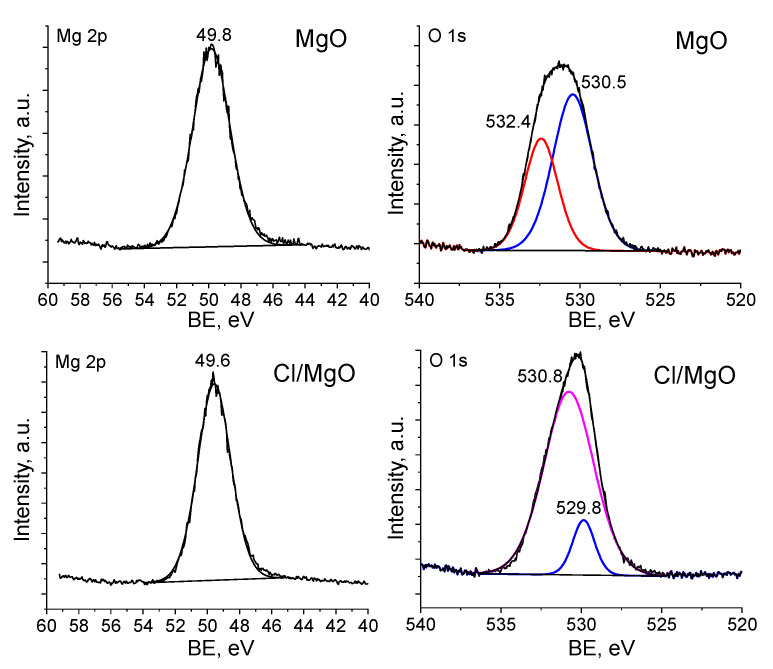
Mg 2p O 1s and Cl 2p XPS spectra of MgO and Cl/MgO.

More light on the way amine anchored on magnesium oxide support can be achieved from XPS investigation. Figure 6 presents the Mg 2p XPS spectra of MgO before and after (3-chloropropyl)trimethoxysilane anchoring. The Mg 2p spectrum of MgO shows one peak at 49.8 eV assigned to magnesium atoms binding with –OH species (Mg-OH). The presence of Mg-OH species was also verified by the peak at 532.4 eV detected in O 1s XPS spectrum of this solid. Moreover, in the O 1s region a peak at 530.5 eV is observed, which can be assigned to the lattice oxygen atoms binding with magnesium atoms, i.e., Mg-O [27]. The position of the O 1s peak attributed to Mg-O was shifted to lower binding energy (529.8 eV) after ClPTMS loading onto MgO. Moreover, the band characteristic of oxygen from Mg-OH species was replaced by another one (BE 530.8) typical of oxygen in Mg-O-Si. The described results proved the presence of interaction between magnesium oxide and chlorine precursor (Scheme 1). At the same time they showed the employment of most Mg-OH species in the anchoring of ClPTMS. This observation is in line with the results coming from FTIR study described above (Figure 4).

**Figure 7:**
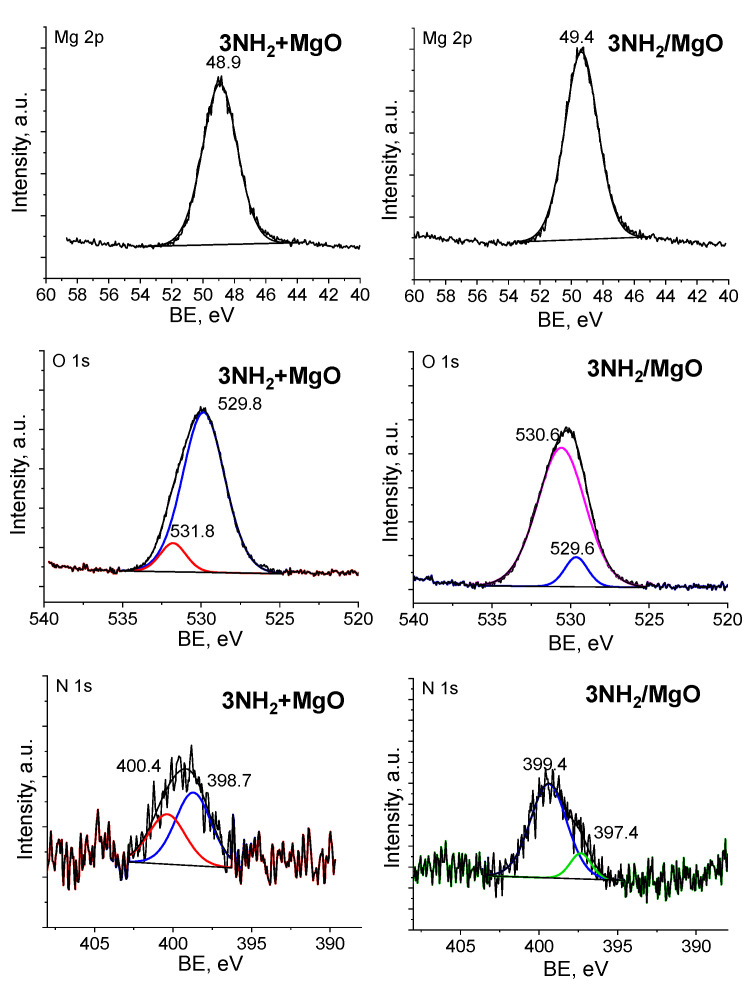
Mg 2p O 1s and Cl 2p XPS spectra of 3NH_2_/MgO and 3NH_2_ + MgO.

The impact of tris(2-aminoethyl)amine on magnesium and oxygen species in MgO is manifested in the XPS spectra shown in Figure 7. As concerns the magnesium spectrum, a shift of binding energy towards lower value of energy in Mg 2p XPS spectrum for both 3NH_2_/MgO (∆BE = 0.4 eV) and 3NH_2_ + MgO (∆BE = 0.9 eV) is observed. This shift was more spectacular for the latter sample, which implies greater impact of TAEA deposition on magnesium species in MgO, if amine was directly loaded into support. In the O 1s region, the changes in the spectra depend on the modification procedure. For the direct modification with TAEA, the band characteristic of oxygen in Mg-OH is shifted from 532.4 eV (for MgO) to 531.8 eV (for 3NH_2_ + MgO) and the one attributed to O 1s BE in Mg-O species is shifted from 530.5 eV to 529.8 eV, which indicate strong interaction between TAEA and both types of oxygen in MgO. When TAEA was anchored on MgO functionalized with ClPTMS (Cl/MgO) both bands characteristic of Mg-O-Si and Mg-OH species were only slightly shifted by 0.2 eV. It confirms the results obtained from the UV–VIS study showing exclusively one form of TAEA species anchored in 3NH_2_/MgO by the bonding with functionalizing agent (ClPTMS) as shown in Scheme 1. 

The N 1s XPS spectrum of 3NH_2_ + MgO (Figure 7) confirms the suggestion of hydrogen bonding of TAEA in this sample, by the presence of a peak at 400.4 eV typical of hydrogen bonded –NH_2_ species (Scheme 1). In addition to this band, a band at 398.7 eV was detected and it was assigned to free –NH_2_ groups [35]. The latter band dominated showing the predominance of free amine groups. The band typical of free amine species at 399.4 eV was also observed in the N 1s XPS spectrum of 3NH_2_/MgO. However, in the spectrum of 3NH_2_/MgO a new band appeared (N 1s BE = 397.4 eV), whose presence is due to nitrogen in the C-N bond between ClPTMS and TAEA. Its presence confirms that amine was mainly homogeneously anchored on MgO modified first with chlorine precursor.

**Figure 8:**
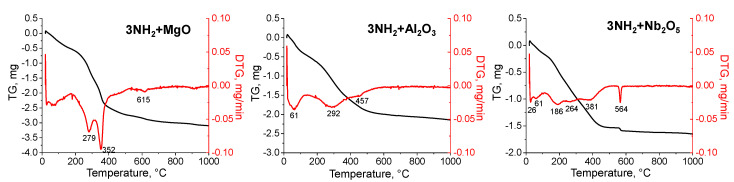
Thermogravimetry analysis of samples modified directly with TAEA.

An important feature of the catalysts modified with tris(2-aminoethyl)amine is the strength of interaction between the modifier and the support as it determines the activity of amine groups. The greater the modifier—support interaction, the lower the interaction of amine groups with the substrates in the Knoevenagel condensation and the lower the activity. This parameter was studied by the thermogravimetric analysis of the hybrid materials prepared by one-step direct TAEA anchoring on metal oxides. The results of these studies are shown in Figure 8 in the form of weight loss (TG and DTG curves) with increasing temperature of heating in air atmosphere. The decomposition of hybrid materials by release of TAEA from the surface of metal oxides led to the loss of weight in the range of 200–500 °C. The DTG curves show the two-step release of TAEA (characteristic of 3NH_2_ + MgO and 3NH_2_ + Al_2_O_3_) due to heterogeneous anchoring of amine on the surface of metal oxides (as indicated by UV–VIS analyses, Figure 5). It is visible that TAEA is more stable on Al_2_O_3_ (stronger held on metal oxide) than on MgO support because the temperatures of TAEA release from alumina is higher. It corroborates the impact of acid-base properties of the support on the strength of interaction between the anchored modifier and the support. The peaks above 500 °C for 3NH_2_ + MgO and 3NH_2_ + Nb_2_O_5_ are due to phase transformation of metal oxides and are not accompanied by a significant loss of mass as seen from TG curves. As concerns niobia support the anchoring of TAEA takes place in a different manner (with participation of BAS and LAS on the metal oxide surface). Therefore, the maxima of temperature of TAEA release cannot be related to the other metal oxide supports.

### 2.3. Knoevenagel Condensation between Furfural and Malononitrile

**Scheme 2:**
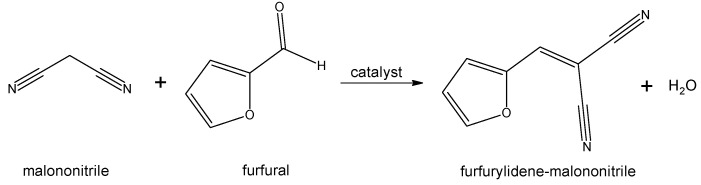
The reaction between malononitrile and furfural.

It has been already reported that Knoevenagel condensation between aldehydes and active methylene compounds proceeds efficiently over catalysts which contain basic amine species [16,36,37,38]. Thus, the use of catalysts containing tris(2-aminoethyl)amine, as a basic modifier, and three different supports (MgO, Al_2_O_3_ and Nb_2_O_5_) showing different acid-base properties for this reaction gave hope to identify a hybrid material effective in the tested catalytic reaction. TAEA contains a higher number of amine species per one molecule, which should accelerate the Knoevenagel condensation, whereas the used supports of different characters can influence the efficiency of tris(2-aminoethyl)amine anchoring, its basicity strength and stability. The reaction between furfural and malononitrile, shown in Scheme 2, was expected to bring some conclusions drawn from the efficiency of TAEA deposition on metal oxides and the interaction between TAEA and support used for catalyst synthesis. 

**Figure 9:**
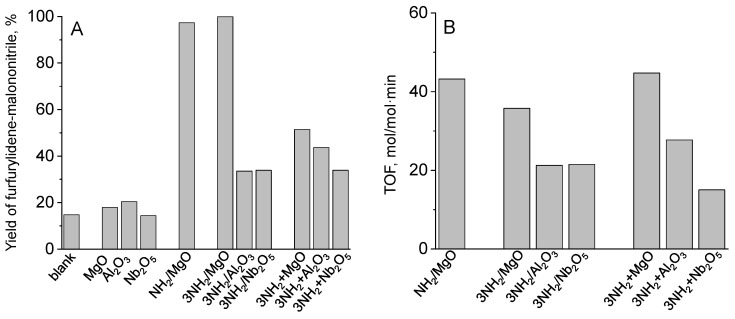
(**A**) the results of the Knoevenagel condensation between furfural and malononitrile performed for 30 min at 60 °C in the presence of synthesized samples. (**B**) activity of catalysts in the condensation between furfural and malononitrile, expressed as turnover frequency (TOF) calculated as moles of furfural converted on one mole of TAEA or APTMS for one minute.

The results of Knoevenagel condensation between furfural and malononitrile presented in Figure 9 show that the anchoring of tris(2-aminoethyl)amine on MgO, Al_2_O_3_ and Nb_2_O_5_ caused an increase in the activity of metal oxides irrespective of the strategy of oxides modification. It is worth noting that the selectivity of tested reaction was 100% to the main product, i.e., furfurylidene-malononitrile. Independently of the modification procedure, TAEA deposited on MgO exhibited much larger activity than the hybrid materials based on Al_2_O_3_ and Nb_2_O_5_. The increase in activity after TAEA loading was especially significant for 3NH_2_/MgO, which can be correlated with a greater amount of TAEA deposited on this material in comparison with that on 3NH_2_ + MgO. However, the catalysts activity did not depend only on the amount of TAEA modifier because the hybrid materials based on Al_2_O_3_ and Nb_2_O_5_ were considerably less active (irrespective of the modification protocol) than those based on MgO although the amount of TAEA deposited on MgO was significantly lower than that on the other two metal oxides. Thus, it is clear that the acid-base properties of metal oxides which determined the type of TAEA anchoring and the strengths of interaction between TAEA and the support (being the lowest for MgO support) are crucial for preparation of hybrid materials effective in Knoevenagel condensation investigated in this work. Thus, the influence of basic support on the catalysts activity has been evidenced. 

As the amount of anchored tris(2-aminoethyl)amine was different for the synthesized samples, a comparison of catalysts activity was made on the basis of the TOF (turnover frequency) calculated as moles of furfural converted per one mole of basic modifier in one minute (Figure 9). Taking into account TOF numbers, the TAEA was the most active when it was loaded onto MgO, especially if the modifier was anchored directly on the support (3NH_2_ + MgO). For this sample tris(2-aminoethyl)amine was anchored in two different types, which can be correlated with a higher activity. 

In order to estimate if tris(2-aminoethyl)amine is more active than monoamine, MgO was modified with (3-aminopropyl)trimethoxysilane (APTMS) containing one amine group per molecule. The amine groups from 3NH_2_/MgO are slightly more active in the Knoevenagel condensation than those from NH_2_/MgO (Figure 9—activity calculated for one mole amine compound). However, it is important to stress that in the case of TAEA it is enough to anchor 2.5 times fewer modifier molecules than APTMS to obtain almost similar activity. Thus, the hybrid materials based on MgO modified with TAEA can be proposed as attractive catalysts for Knoevenagel condensation between furfural and malononitrile. 

## 3. Materials and Methods

### 3.1. MgO, Al_2_O_3_ and Nb_2_O_5_ Modification Direct with Tris(2-aminoethyl)amine or after (3-chloropropyl)trimethoxysilane (ClPTMS) Anchoring

The modification of the supports with tris(2-aminoethyl)amine was performed by two strategies, i.e., by direct deposition of amine or by its deposition after (3-chloropropyl)trimethoxysilane (ClPTMS) modification, as shown in Scheme 1. The commercially available metal oxides were used for the synthesis of catalysts: MgO from Sigma-Aldrich, St. Louis, MO, USA, Al_2_O_3_ from SASOL, Johannesburg, RPA, and Nb_2_O_5_ from CBMM, Araxá, Minas Gerais, Brazil. Both procedures of metal oxides modification with amine species are described below.

#### 3.1.1. The Direct Modification of Metal Oxides with Tris(2-aminoethyl)amine

Portions of 3 g of each metal oxide studied (first dried overnight at 100 °C) were put into a glass flask. Then 90 cm^3^ of dried toluene and 0.29 cm^3^ of tris(2-aminoethyl)amine (Aldrich, St. Louis, MO, USA) were introduced. The mixtures were stirred and heated at 110 °C for 48 h. The solid product was then filtered off, washed with 160 cm^3^ of toluene and finally dried at 100 °C for 24 h. The samples obtained by this procedure are denoted as 3NH_2_ + MgO, 3NH_2_ + Al_2_O_3_ and 3NH_2_ + Nb_2_O_5_.

#### 3.1.2. The Modification of Metal Oxides with tris(2-aminoethyl)amine after (3-chloropropyl)trimethoxysilane (ClPTMS) Anchoring 

Portions of 4 g of each metal oxide studied (first dried overnight at 100 °C) were put into a glass flask. Then 150 cm^3^ of dried toluene and 4 cm^3^ of ClPTMS (Aldrich, St. Louis, MO, USA) were introduced. The obtained mixtures were stirred for 15 min at room temperature and then heated at 110 °C for 24 h. The solid product was then filtered, washed with 45 cm^3^ of dried toluene and dried at 100 °C for 24 h.

The anchoring of tris(2-aminoethyl)amine to the supports functionalized with ClPTMS was performed as follows. A portion of 2.3 cm^3^ of tris(2-aminoethyl)amine (Aldrich, St. Louis, MO, USA) and 4.5 cm^3^ of triethylamine (added for neutralization of HCl formed during the metal oxide modification with amine as a result of reaction between amine species with anchored ClPTMS) was introduced to the mixture containing 3 g of metal oxide functionalized first with ClPTMS (dried overnight at 100 °C) and 90 cm^3^ of dried toluene. The obtained mixture was heated at 110 °C for 48 h. The solid product was then filtered off, washed with 160 cm^3^ of toluene and finally dried at 100 °C for 24 h. The obtained samples are denoted as 3NH_2_/MgO, 3NH_2_/Al_2_O_3_ and 3NH_2_/Nb_2_O_5_.

Moreover, a modifier was synthesized as a result of the reaction between ClPTMS and tris(2-aminoethyl)amine. A portion of 4 cm^3^ of ClPTMS was mixed with 2.3 cm^3^ of tris(2-aminoethyl)amine and 90 cm^3^ of toluene. The obtained mixture was then stirred at 110 °C for 48 h. The yellow solid product was then filtered off, washed with 160 cm^3^ of dried toluene and dried at 100 °C for 24 h. The obtained modifier was denoted as 3NH_2_ + Cl.

### 3.2. MgO Modification with (3-Aminopropyl)trimethoxysilane

A portion of 2 g of MgO (first dried overnight at 100 °C) was put into a glass flask. Then 200 cm^3^ of dried toluene and 2.5 cm^3^ of (3-aminopropyl)trimethoxysilane (Aldrich, St. Louis, MO, USA) were introduced. The synthesis mixture was then stirred and heated at 110 °C for 18 h. The solid product was then filtered off and washed with 300 cm^3^ of toluene and finally dried at 80 °C for 24 h. The sample obtained is denoted as NH_2_/MgO.

### 3.3. Samples Characterization

The obtained catalysts were characterized by nitrogen adsorption/desorption, XRD (X-ray diffraction, Bruker, Karlsruhe, Germany), elemental analysis, XPS (X-ray photoelectron spectroscopy, Specs, Berlin, German), FTIR (Fourier-transform infrared spectroscopy, Bruker, Poznan, Poland), UV–Vis (ultraviolet-visibly spectroscopy, Candela, Warszawa, Poland), and thermogravimetric analysis.

N_2_ adsorption/desorption isotherms were measured using an ASAP 2020 instrument. Prior to analysis the samples were outgassed under vacuum at 120 °C for 10 h. The surface area of solids was calculated by the BET method, while the pore volume and diameter were estimated according to the BJH approximation.

XRD patterns were obtained on a Bruker AXS D8 Advance apparatus using CuKα radiation (γ = 0.154 nm), with a step of 0.05° in the wide-angle range (from 21° to 60° of 2 theta). 

Elemental analysis was performed with Elemental Analyzer Vario EL III.

X-ray photoelectron spectroscopy measurements (XPS) were carried out on an Ultra High Vacuum (UHV) System (Specs, Berlin, Germany) equipped with a monochromatic microfocused Al Kα X-ray source (1486.6 eV). Binding energies were referenced to the C 1s peak at 284.6 eV.

Fourier-transform infrared spectra were acquired with a Vertex 70 spectrometer in the range from 4000 cm^−1^ to 400 cm^−1^. Firstly, the mixture of 0.2 g of KBr (Aldrich) and 0.001 g of material was pressed under low pressure into a thin circular pill and immersed into a special cell. Then the FTIR spectrum of the catalyst was recorded. 

The UV–VIS spectra in the range between 800–190 nm were recorded using a Varian-Cary 300 Scan UV–VIS spectrophotometer and spectralon as a reference sample. Before measurements the solids were dried overnight at 100 °C. The spectrum of tris(2-aminoethyl)amine was recorded using a quartz cell (1 cm^3^). Methanol was used as a reference.

Thermogravimetric measurements were made in air atmosphere using a SETARAM SETSYS-12 apparatus in the temperature range 20–1000 °C with a temperature ramp of 5 °C/min. 

### 3.4. Test Reactions

#### 3.4.1. 2-Propanol Dehydration and Dehydrogenation

Decomposition of 2-propanol was carried out using a microcatalytic pulse reactor containing granulated catalyst bed (0.5 < ø < 1 mm, 0.1 g). The reactor was connected to a column (5% Carbowax 20 m) of an SRI 310 chromatograph equipped with a FID detector used for analysis of reaction products. Prior to the reaction the catalyst was activated at 400 °C for 2 h under nitrogen flow (40 cm^3^/min). Then, the 3 μL pulses of 2-propanol (POCH, Polskie Odczynniki Chemiczne, Lublin, Poland) after introduction into the reactor were vaporized and passed through the catalyst bed under nitrogen flow, 65 cm^3^/min. The reaction was studied in the temperature range from 150 °C to 300 °C.

#### 3.4.2. Dehydration and Cyclization of 2,5-Hexanedione

A portion of granulated catalyst bed (0.5 < ø < 1 mm, 0.05 g) placed in the reactor was activated for 2 h at 400 °C under nitrogen flow (40 cm^3^/min). Then, 0.5 cm^3^ of 2,5-hexanedione (2,5-HDN), dosed with a pump system equipped with a syringe, was passed continuously through the catalyst bed as a vapour, at 350 °C for 30 min with the flow of nitrogen used as a carrier gas (40 cm^3^/min). The reaction products were collected into a cold trap (liquid nitrogen + 2-propanol) and analysed by gas chromatograph SRI 310C (DB-1 column 30 m) equipped with a TCD detector under helium as a carrier gas.

### 3.5. Knoevenagel Condensation

All catalysts were tested in Knoevenagel condensation carried out without any solvent (Scheme 2). A mixture of furfural (20 mmol, 1.92 g) (Aldrich, St. Louis, MO, USA) and malononitrile (20 mmol, 1.32 g) (Aldrich, St. Louis, MO, USA) together with 30 mg of the catalyst (preliminary dried overnight at 100 °C) was placed in a quartz reactor in EasyMax system and heated at 60 °C upon vigorous stirring. The reactions were performed for 30 min. Products were analysed by a gas chromatograph (Thermo Scientific, Waltham, MA, USA) equipped with a 60 m VF-5 ms capillary column and FID detector.

## 4. Conclusions

The catalysts based on hybrid materials containing MgO, Al_2_O_3_ and Nb_2_O_5_ as supports and tris(2-aminoethyl)amine as an active ingredient were synthesized and characterized in details. The TAEA was anchored directly on metal oxides or after functionalization with CIPTMS. The metal oxides exhibited different acid-base nature, thus, they showed impact on differential effectiveness of TAEA loading and the strength of TAEA-support interaction as well as catalysts activity in Knoevenagel condensation between furfural and malononitrile.

The use of Al_2_O_3_ and Nb_2_O_5_ as supports allowed incorporation of a larger amount of tris(2-aminoethyl)amine species due to acidic centres present on the surface of both metal oxides. In the direct one-step modification of metal oxide with TAEA, the amount of amine deposited on Nb_2_O_5_ was greater than that on Al_2_O_3,_ because in the first metal oxide BAS and LAS were involved in the anchoring of TAEA. The formation of two different types of TAEA species on MgO and Al_2_O_3_ (i.e., metal oxides containing basic hydroxyls) was evidenced for the samples prepared by one-step amine anchoring (single and double coordinated species). The acid-base properties of both metal oxides determined the strength of TAEA-support interaction, which was much lower for 3NH_2_ + MgO hybrid material. In contrast to the samples prepared by the one-step procedure, in the materials obtained by the two-step modification TAEA was not directly bonded to the surface of metal oxide and therefore, on MgO and Al_2_O_3_ supports homogeneous anchoring of TAEA via interaction with ClPTMS functionalization agent was estimated. For Nb_2_O_5_ support two different species were postulated to be formed after TAEA anchoring on ClPTMS functionalized niobia, one anchored to ClPTMS bonded to the solid surface and the second formed in the interaction between LAS (niobium cations) on niobia surface and TAEA.

The catalytic activities of the hybrid materials obtained were examined in the Knoevenagel condensation between furfural and malononitrile. A superior activity was observed for the catalysts containing MgO as a support in which TAEA—metal oxide interaction was the weakest. The activity expressed by TOF was much higher for 3NH_2_ + MgO than 3NH_2_/MgO suggesting that the introduction of additional functionalizing agent (ClPTMS) containing methoxy and chlorine species lowered the basicity strength of amine groups.

## Figures and Tables

**Table 1:** The results of 2,5-hexanedione (2,5-HDN) cyclization at 350 °C.

Catalyst	Conversion of 2,5-HDN (%)	Selectivity (%)		MCP/DMF
		MCP	DMF	
MgO	89 ± 0.4	0.4 ± 0.01	99.6 ± 0.01	249
Al_2_O_3_	36 ± 0.2	7.0 ± 0.1	93.0 ± 0.1	13
Nb_2_O_5_	8 ± 0.1	65.9 ± 0.9	34.1 ± 0.9	0.5

**Table 2:** Nitrogen content in the catalysts estimated from elemental analysis.

Catalyst *	Amine Modifer, mmol/g (Nominal)
3NH_2_ + MgO	0.25
3NH_2_ + Al_2_O_3_	0.35
3NH_2_ + Nb_2_O_5_	0.50
NH_2_/MgO	0.47
3NH_2_/MgO	0.62
3NH_2_/Al_2_O_3_	1.40
3NH_2_/Nb_2_O_5_	1.40

* 3NH_2_ + metal oxide—TAEA deposited directly on oxides; NH_2_/metal oxide—APTMS anchored directly on oxide; 3NH_2_/metal oxide—TAEA deposited on oxides after ClPTMS functionalization.

**Table 3:** The texture/structure parameters of the modified metal oxides.

Catalyst	Surface Area, m^2^/g	Pore Volume, cm^3^/g	Average Pore Diameter, nm (BJH)	Average Pore Diameter, nm (PSD)
MgO	130.0 ± 0.9	0.29	7.86	5.9
Al_2_O_3_	172.0 ± 1.3	0.47	8.49	10.2
Nb_2_O_5_	123.0 ± 1.1	0.12	5.70	4.6/macropores
3NH_2_ + MgO	50 ± 0.5	0.17	13.26	5.9
3NH_2_ + Al_2_O_3_	146 ± 1.9	0.40	7.79	9.6
3NH_2_ + Nb_2_O_5_	40 ± 0.4	0.09	8.52	4.6/macropores
3NH_2_/MgO	69.9 ± 1.2	0.20	8.92	5.9
3NH_2_/Al_2_O_3_	29.3 ± 0.4	0.08	9.16	9.6
Cl/Nb_2_O_5_	71.0 ± 0.8	0.08	7.40	4.6/macropores
3NH_2_/Nb_2_O_5_	0.4 ± 0.01	0.0004	10.4	-
